# An interpretable stacking ensemble learning framework based on multi-dimensional data for real-time prediction of drug concentration: The example of olanzapine

**DOI:** 10.3389/fphar.2022.975855

**Published:** 2022-09-27

**Authors:** Xiuqing Zhu, Jinqing Hu, Tao Xiao, Shanqing Huang, Yuguan Wen, Dewei Shang

**Affiliations:** ^1^ Department of Pharmacy, The Affiliated Brain Hospital of Guangzhou Medical University, Guangzhou, China; ^2^ Guangdong Engineering Technology Research Center for Translational Medicine of Mental Disorders, Guangzhou, China; ^3^ Department of Clinical Research, Guangdong Second Provincial General Hospital, Guangzhou, China

**Keywords:** olanzapine, drug concentration, therapeutic drug monitoring, stacking, machine learning, electronic health record, interpretability, model-informed precision dosing

## Abstract

**Background and Aim:** Therapeutic drug monitoring (TDM) has evolved over the years as an important tool for personalized medicine. Nevertheless, some limitations are associated with traditional TDM. Emerging data-driven model forecasting [e.g., through machine learning (ML)-based approaches] has been used for individualized therapy. This study proposes an interpretable stacking-based ML framework to predict concentrations in real time after olanzapine (OLZ) treatment.

**Methods:** The TDM-OLZ dataset, consisting of 2,142 OLZ measurements and 472 features, was formed by collecting electronic health records during the TDM of 927 patients who had received OLZ treatment. We compared the performance of ML algorithms by using 10-fold cross-validation and the mean absolute error (MAE). The optimal subset of features was analyzed by a random forest-based sequential forward feature selection method in the context of the top five heterogeneous regressors as base models to develop a stacked ensemble regressor, which was then optimized *via* the grid search method. Its predictions were explained by using local interpretable model-agnostic explanations (LIME) and partial dependence plots (PDPs).

**Results:** A state-of-the-art stacking ensemble learning framework that integrates optimized extra trees, XGBoost, random forest, bagging, and gradient-boosting regressors was developed for nine selected features [i.e., daily dose (OLZ), gender_male, age, valproic acid_yes, ALT, K, BW, MONO#, and time of blood sampling after first administration]. It outperformed other base regressors that were considered, with an MAE of 0.064, R-square value of 0.5355, mean squared error of 0.0089, mean relative error of 13%, and ideal rate (the percentages of predicted TDM within ± 30% of actual TDM) of 63.40%. Predictions at the individual level were illustrated by LIME plots, whereas the global interpretation of associations between features and outcomes was illustrated by PDPs.

**Conclusion:** This study highlights the feasibility of the real-time estimation of drug concentrations by using stacking-based ML strategies without losing interpretability, thus facilitating model-informed precision dosing.

## 1 Introduction

As the foundation of personalized medicine, traditional therapeutic drug monitoring (TDM) has multiple advantages, including the simple interpretation of results and intuitive dosing adjustments by using the “direct or inverse rule of three,” or dosing nomograms (Guo et al., 2013). However, its implementation commonly requires waiting for a steady state, and might be hindered owing to a lack of access to dedicated staff, expensive equipment, and analytical methods (Guo et al., 2013; Leung et al., 2019; Zhu et al., 2021b). Moreover, the test results are influenced by the sampling time, dosage regimens, and covariates, and the possible delayed return of measurements may also raise barriers to TDM (Guo et al., 2013; Leung et al., 2019; Zhu et al., 2021b). Model-informed precision dosing (MIPD) is an emerging, integrative term that is defined as the use of mathematical models to individualize predictions and streamline the TDM process by integrating multi-level patient-specific data (Darwich et al., 2021). It provides quantitative decision support to clinicians for personalized dosing adjustment to improve the outcomes of drug treatment in patients. A typical application of MIPD involves a variety of approaches to modeling, such as pharmacometric models of biology, pharmacology, and physiology. Emerging data-driven approaches, such as Artificial Intelligence (AI), have attracted considerable attention in recent years (Ribba et al., 2020).

Machine learning (ML) is a subfield of AI, and ML algorithms can be roughly classified into three categories: 1) Supervised, 2) unsupervised, and 3) those based on reinforcement learning. In principle, the role of ML tools in MIPD has two notable aspects. One is that they can partner with other MIPD tools (e.g., pharmacometrics) to significantly reduce computational cost, e.g., by applying ML as an alternative, initial step for covariate screening in population pharmacokinetic (popPK) analysis (Koch et al., 2020). In case of large datasets or complex models, it can also greatly enhance the efficiency of the selection of the popPK model (Sibieude et al., 2022). A previous study has reported that an ensemble model that integrates artificial neural networks and non-linear mixed-effects modeling (NONMEM) generates more powerful predictions than either method (Poynton et al., 2009). Furthermore, owing to the potential roles of ML and AI in connecting big data to pharmacometrics (McComb et al., 2022), these combined approaches have been used to accurately estimate pharmacokinetic parameters (e.g., drug exposure and drug clearance) (Tang et al., 2021; Woillard et al., 2021). Nevertheless, various complex mathematical models for drugs and diseases generally need to be understood and chosen to use these methods, where this may render the modeling process laborious and time consuming (Zhu et al., 2021a). Besides, the model validation methods in ML are not routinely used during the development of pharmacometrics models (McComb et al., 2022), which may hinder the integration of ML and pharmacometrics. The other notable aspect of the role of ML in MIPD is the straightforward prediction of drug concentration or drug dose. This enables simple individualized treatment by changing such influential factors as the dose or dosing intervals. A previous study used the extra trees-based regression algorithm to establish a predictive model for dose-adjusted concentrations of lamotrigine (Zhu et al., 2021a). Guo et al. (2021) established an eXtreme gradient boosting (XGBoost) model for the prediction of the active moiety of risperidone in the next TDM based on the initial TDM. Both of these studies evaluated a variety of ML models. Even though different types of regression algorithms are available for such ML tasks, individual regressors may deliver similar or even identical performance to them. Besides, a major drawback of the single model-based approach is its instability, which can degrade performance and induce a larger bias compared with ensemble methods, which improve performance by combining diverse forecasts from multiple models (Bourel and Segura, 2018; Kalagotla et al., 2021).

The aforementioned limitations have motivated researchers to develop novel ensemble methods, which can be classified into two types, i.e., homogeneous and non-homogeneous methods (Bourel et al., 2017). Homogeneous ensemble methods, such as the random forest (RF) (Breiman, 2001), bagging (Breiman, 1996), and boosting (Freund and Schapire, 1997; Schapire et al., 1998), combine single-type base learning algorithms to build homogeneous base learners. Non-homogeneous methods (also called heterogeneous ensemble methods or consensus methods) combine multiple models with different natures, like stacking (i.e., meta-ensembling) (Wolpert 1992; Bourel et al., 2017), and each base model is built upon the same training data. In this paper, we restrict ourselves to stacking with stratified five-fold cross-validation. It has the advantage of a meta-learner that combines the outputs of base learners as new input features for training and testing, where this improves the predictive performance of these joint models (Kalagotla et al., 2021).

Olanzapine (OLZ) is a commonly prescribed second-generation antipsychotic for the treatment of psychotic conditions (e.g., schizophrenia and bipolar disorder). The unlicensed or off-label use of OLZ is common in pediatrics (Zhu et al., 2018). The large inter-individual pharmacokinetic variations in OLZ, which are influenced by genetic and environmental factors (Arnaiz et al., 2021), make TDM an essential part of personalizing OLZ treatment in any age group, especially the pediatric population (high-level recommendation to use TDM according to guidelines of the *Arbeitsgemeinschaft für Neuropsychopharmakologie und Pharmakopsychiatrie* consensus) (Hiemke et al., 2018). Several studies, using popPK and multiple regression analyses, have been conducted to reveal the sources of the pharmacokinetic variability of OLZ by integrating various covariates (e.g., age, gender, body weight, and concomitant medications) (Deng et al., 2020; Xiao et al., 2021; Zang et al., 2021). All of these studies involved low-dimensional data, with a small sample size and few patient-related variables. By taking OLZ as an example in this work, we preliminarily mine the possible relevant covariates based on a TDM-OLZ dataset derived from real-world multi-dimensional electronic health records (EHRs) for pharmacometrics analysis. We use this to develop an MIPD tool—the state-of-the-art interpretable stacking ensemble learning framework—that integrates different types of base learners for the accurate, real-time estimation of drug concentrations.

## 2 Materials and methods

### 2.1 Brief introduction to the proposed approach

**FIGURE 1:**
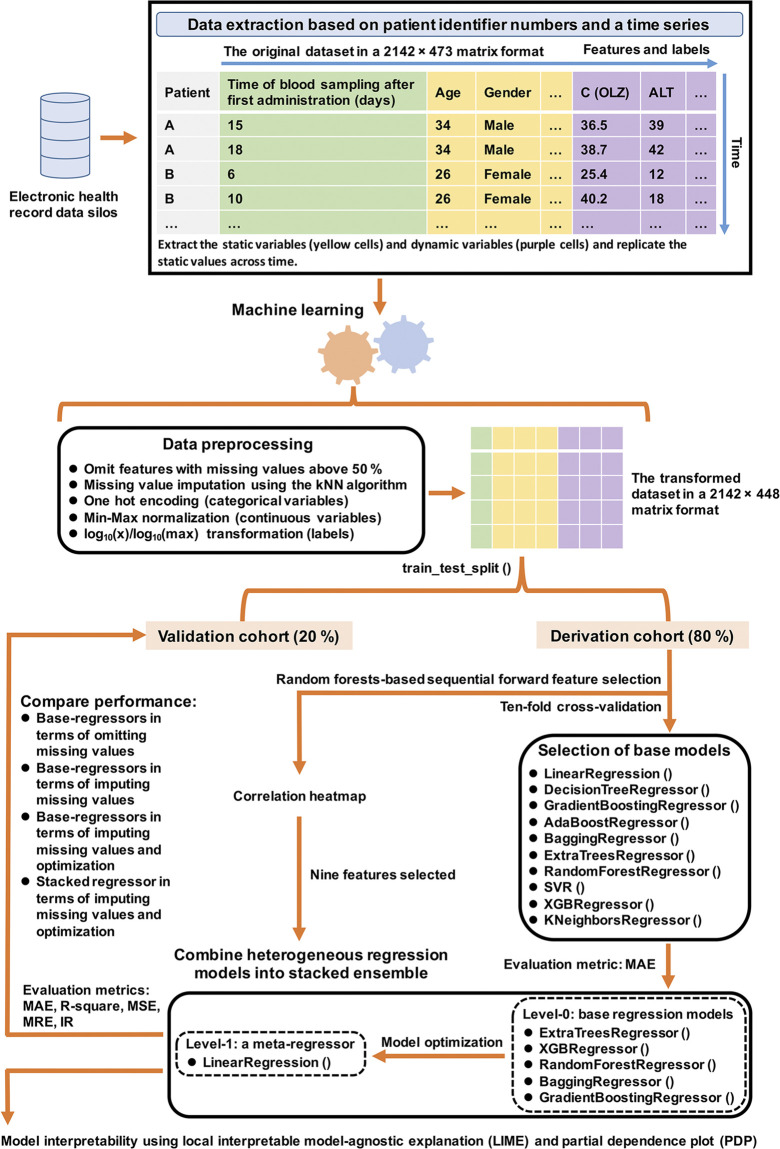
Flowchart of this study.

Figure 1 illustrates the flowchart of this work. We acquired the EHR data to construct the original dataset (i.e., the TDM-OLZ dataset) during the TDM of OLZ of 927 patients, who had received OLZ treatment in the latter half of 2018, at the Affiliated Brain Hospital of Guangzhou Medical University in Guangzhou, China. We then conducted data preprocessing, and chose the base regression model as well as the relevant features based on the derivation cohort. Following this, we compared the overall predictive performance of the stacked regressor and base regressors in the context of omitting and imputing missing values as well as model optimization by using multiple evaluation metrics for the validation cohort. Finally, we provide explanations of the model at the individual and the feature levels.

### 2.2 Original dataset

The original dataset consisted of features (i.e., patient-specific attributes) and labels [i.e., the TDM measurements of OLZ; abbreviation: C (OLZ)]. Their values were extracted based on patient identifier numbers and a time series of TDM blood sampling after OLZ was first administered. Some features were static variables, which are normally declared as constants during hospitalization (e.g., gender and genotypes), whereas others were dynamic variables that were susceptible to substantial changes over time [e.g., alanine transaminase (ALT) and C (OLZ)].

The serum concentration of OLZ was determined by using a previously reported analytical method developed by our laboratory, where the calibration curve was linear over a wide range of concentrations of 2–200 ng/ml (Xiao et al., 2021). In particular, we replicated the static values across time, excluded OLZ concentrations below the lower limit of quantification, and assumed that TDM measurements of other medications were zero if they had not been co-administrated. This finally yielded an original dataset of 2,142 × 473 matrices, containing 2,142 TDM measurements of OLZ and 472 features (Figure 1). The retrospective data collection was approved by the Ethics Committee of the Affiliated Brain Hospital of Guangzhou Medical University ([2021] NO.027).

### 2.3 Data preprocessing

Before using the data for training, data preprocessing (i.e., appropriately scaling and transforming the entire dataset) was performed to obtain high-quality data containing as much useful information as possible to facilitate training and testing. The steps of preprocessing were as follows:

Step 1 : omitting features with more than 50% missing values.

It was inevitable that some values of the samples for some features in the dataset derived from the EHR system were missing. One strategy to address this issue is to remove the features with missing values. However, this might lead to the removal of a large number of rich features and the loss of valuable information for ML algorithms. In this study, we included all features that occurred in at least 50% of the samples (Meyer et al., 2018).

Step 2 : missing value imputation by using the k-nearest neighbor (kNN) method.

Another means of dealing with those missing data is kNN imputation (Beretta and Santaniello, 2016). In this work, we used the 3NN schema to find the three neighbors closest to the query points, and then imputed them based on their weighted mean values. In this case, the closer the neighbor of a query point was to it, the greater was its weight. The distance metric used was Minkowski distance, and is defined as:

$${\left(\overset{n}{\underset{i=1}{\sum }}{\left|x_{i}-y_{i}\right|}^{p}\right)}^{1/p}$$

where p is the power parameter for the Minkowski metric. We used its default value of two, which is equivalent to using the Euclidean distance.

Step 3 : one-hot encoding of categorical variables.

Converting ordinal categorical features into numerical values is commonly required for most ML algorithms, whereas nominal categorical features should be handled by using the one-hot encoding technique. In this technique, new variables (also known as dummy variables) are created for every nominal categorical feature, while the number of newly created dummy variables depends on the number of categories present. This technique was applied to all categorical features in our original dataset. After performing one-hot encoding, we dropped the first category for each feature because one of the categories could be generated from the others, and hence no more information could be added to the modeling process if this extra category was retained.

Step 4 : Min–max normalization on continuous variables.

The generalization performance and stability of ML models are highly dependent on the quality of the input data (Rahman et al., 2022). We used min–max rescaling to normalize the continuous feature variables to the range [0, 1] *via* the following formula:

$$x^{\prime }=\left(x-x_{\min}\right)/\left(x_{\max}-x_{\min}\right)$$

where $x_{\min}$ and $x_{\max}$ denote the minimum and maximum values of the feature, respectively.

Step 5 : log transformation of labels.

Given the wide range of OLZ measurements, we used the log transformation of the labels to make them more symmetric, or to render their distribution similar to a normal distribution, to ensure reliable and stabilized numerical forecasts. This method transforms the data to the range (0, 1] through the following formula:

$$y^{\prime }=\log_{10}\left(y\right)/\log_{10}\left(y_{\max}\right)$$

where $y_{\max}$ denotes the maximum value of the label.

### 2.4 Selecting the base regression model

#### 2.4.1 Splitting dataset

A transformed dataset in 2,142 × 448 matrix format was generated after data preprocessing (Figure 1). The number of features decreased to 447. Subsequently, 80% of the samples (1,713 measurements) from the transformed dataset were randomly selected as the “derivation cohort” for model construction and feature selection, and the remaining 20% (429 measurements) were used as the “validation cohort” for testing.

#### 2.4.2 Selection method

We used 10-fold cross-validation to compare the performance of 10 candidate regression models with default hyperparameters in the derivation cohort by using the mean absolute error (MAE), i.e., the average of the absolute value of the residuals, calculated as (Zhu et al., 2021a):

$$MAE=\frac{1}{n}\;\overset{n}{\underset{i=1}{\sum }}\left|y_{i}-{\hat{y}}_{i}\right|$$

where $y_{i}$ and ${\hat{y}}_{i}$ denote the predicted and measured values, respectively. The five best-performing models were chosen as the base regressors to establish the stacking-based model of prediction.

#### 2.4.3 Candidate models

All models, apart from the multiple linear regression (MLR) model used as a reference, were non-linear. They included extra trees-based regression (ETR), random forest regression (RFR), bagging regression (BR), gradient-boosted regression (GBR), AdaBoost regression (ABR), XGBoost regression (XGBR), support vector regression (SVR), k-nearest neighbor regression (KNR), and decision tree regression (DTR). Non-linear models have been shown to be suitable for non-linear and dynamic states of diseases because of the inevitable noise contained in the relevant datasets (Zhou et al., 2019; Zhu et al., 2021a). A brief description of each of the candidate models is given in Table 1.

**TABLE 1:** Brief descriptions of 10 candidate regression models, including the related packages and their parameters (default settings).

Model	Package	Key hyperparameters
ETR	scikit-learn 0.23.2 (from sklearn.ensemble import ExtraTreesRegressor)	“n_estimators”: 100, “max_depth”: None, “min_samples_leaf”: 1, ‘”min_samples_split”: 2, “max_features: auto”
RFR	scikit-learn 0.23.2 (from sklearn.ensemble import RandomForestRegressor)	“n_estimators”: 100, “max_depth”: None, “min_samples_leaf”: 1, “min_samples_split”: 2, ‘”max_features”: auto
BR	scikit-learn 0.23.2 (from sklearn.ensemble import BaggingRegressor)	“n_estimators”: 10, “max_depth”: 1.0, “max_samples”: 1.0
GBR	scikit-learn 0.23.2 (from sklearn.ensemble import GradientBoostingRegressor)	“n_estimators”: 100, “max_depth”: 3, “min_samples_leaf”: 1, “min_samples_split”: 2, “alpha”: 0.9, “learning_rate”: 0.1, “max_features”: None
ABR	scikit-learn 0.23.2 (from sklearn.ensemble import AdaBoostRegressor)	“n_estimators”: 50, “loss”: linear, “learning_rate”: 1.0
XGBR	xgboost 1.3.3 (from xgboost import XGBRegressor)	“n_estimators”: 100, “max_depth”: None, “min_child_weight”: None, “gamma”: None, “colsample_bytree”: None, “subsample”: None, “reg_alpha”: None, “reg_lambda”: None, “learning_rate”: None
SVR	scikit-learn 0.23.2 (from sklearn.svm import SVR)	“C”: 1.0, “gamma”: scale, “epsilon”: 0.1, “kernel”: rbf
KNR	scikit-learn 0.23.2 (from sklearn.neighbors import KNeighborsRegressor)	“weights”: uniform, “n_neighbors”: 5, “p”: 2
DTR	scikit-learn 0.23.2 (from sklearn.tree import DecisionTreeRegressor)	“criterion”: squared_error, “max_depth”: None, “min_samples_leaf”: 1, “min_samples_split”: 2, “max_features”: None
MLR	scikit-learn 0.23.2 (from sklearn.linear_model import LinearRegression)	“fit_intercept”: True, “normalize”: deprecated, “n_jobs”: None, “copy_X”: True

Given that the ensemble technique usually yields better performance than a single model (e.g., SVR, KNR, DTR, and MLR) (Yin et al., 2021), we provide a brief introduction to the candidate models based on popular ensemble learning techniques, including bagging and boosting. Unlike the stacking technique that uses heterogeneous base learners, bagging (also known as bootstrap aggregation, proposed by Breiman, 1996) involves fitting multiple distinct decision trees in parallel on numerous subsets (bags) of the same training dataset by using bootstrapping-based sampling techniques. The final predictions are obtained through the average value and voting for regression and classification, respectively. This can yield substantial gains in predictive performance and robustness through a reduction in the variance of the models (i.e., avoiding overfitting). Some ML algorithms use bagging techniques, such as ETR, RFR, and BR. In boosting, on the contrary, all individual models are built serially. That is, it is a sequential ensemble method involving the sequential addition of learning models that correct for the errors made by preceding models, and outputting a weighted average of the predictions of the individual learners (Yaman and Subasi, 2019). One benefit of using the boosting technique is that it can simultaneously reduce the bias and variance of the model (Cao et al., 2010). Some commonly used ML algorithms have been developed based on the basic idea of boosting, including GBR, ABR, and XGBR (Freund and Schapire, 1997; Dal Molin Ribeiro and Coelho, 2020).

#### 2.4.4 K-fold cross-validation

Cross-validation is commonly used to assess the performance of learners (i.e., their capability of generalizing new and unseen data) and compare models. K-fold cross-validation is a typical cross-validation strategy, where K is any number (Kalagotla et al., 2021). We used *K* = 10. The process of K-fold cross-validation was straightforward:

Step 1: The samples in the derivation cohort were randomly divided into K folds (subsets), and this process was iterated K times.

Step 2: For each iteration, the model was tested on the *K*th fold of the dataset (i.e., the test set) while the remaining K—1 folds (i.e., the training set) were used for training.

Step 3: Step 2 was repeated until each of the K folds had served as the test set.

Step 4: The average cross-validation score in the test set across all K folds was taken as the final result to compare the models.

### 2.5 Random forest-based sequential forward feature selection

The proposed random forest-based sequential forward feature selection was implemented as detailed below (Pan et al., 2017; Yao et al., 2020; Zhu et al., 2021a).

#### 2.5.1 Measure of feature importance based on random forest

The features were ranked in descending order of importance scores of the corresponding impurity-based variable obtained by the random forest algorithm. The importance of each variable was computed as the normalized total reduction in the criterion (i.e., impurity function) due to it. If the decrease was large, the variable was considered important, and vice versa. The reduction in the variance of the node samples, which determines the impurity of the regression node, was selected as the criterion of feature selection for regression. The following equations were used to obtain the normalized feature importance (Akter et al., 2021):

1) Impurity:

$$G\left(m\right)=\frac{1}{N_{m}}\underset{i\in N}{\sum }{\left(Y_{i}-{\bar{Y}}_{m}\right)}^{2}$$

where $N_{m}$ and ${\bar{Y}}_{m}$ denote the sample size and the sample mean of the current node m , respectively.

2) Assuming that a tree T is split at node k . The reduction in impurity after splitting k into left and right daughter nodes $k_{left}$ and $k_{right}$ under a proposed split for a feature is:

$$n_{k}=p_{k}G_{k}-p_{k_{left}}G_{k_{left}}-p_{k_{right}}G_{k_{right}}$$

where $p_{k}$ , $p_{k_{left}}$ , and $p_{k_{right}}$ are the ratios of observations in nodes k , $k_{left}$ , and $k_{right}$ , respectively, while $G_{k}$ , $G_{k_{left}}$ , and $G_{k_{right}}$ are the impurities of nodes k , $k_{left}$ , and $k_{right}$ , respectively.

3) Importance of feature i :

$$f_{i}=\frac{\sum_{j\in nodes\;split\;on\;feature\;i\;}n_{j}}{\sum_{k\in all\;nodes\;}n_{k}}$$

4) Normalized importance of feature i in tree T :

$${normf}_{i}=\frac{f_{i}}{\sum_{j\in all\;features\;}f_{j}}$$

5) Importance of feature i calculated in all trees $T_{total}$ :

$${RFf}_{i}=\frac{\sum_{j\in all\;trees\;}{normf}_{ij}}{T_{total}}$$

#### 2.5.2 Sequential forward feature selection

To address the arbitrariness of the selection of the feature subset and the instability of the results, we used sequential forward selection, a well-known wrapper-based approach, to generate the optimal feature subset (Yao et al., 2020). Feature selection based on sequential forward selection is a bottom-to-top search method that starts with an empty feature set. At each sequential step, the system adds the most important feature selected according to a given criterion to form a new feature subset (Marcano-Cedeño et al., 2010; Yao et al., 2020). The relative feature importance obtained from random forest was used as the evaluation criterion. In each subsequent iteration, the pros and cons of the generated feature subset were evaluated according to the performance of the regressor on the test set by using 10-fold cross-validation and the MAE. The features sequentially added should increase the score on the performance metric. The optimal feature subset was considered to have been established when “no considerable decline” in the MAE was observed.

### 2.6 Correlation analysis

The following two aims were considered: 1) To investigate the association among the features to remove collinearly multiple correlated features; and 2) to explore the positive or negative relationships between the predictors and the labels.

We used the Pearson correlation coefficient, among the most commonly used correlation coefficients, to calculate the linear relationship between the given variables. It is expressed as follows:

$$R\left(x,y\right)=\frac{\sum_{i=1}^{n}\left(x_{i}-\bar{x}\right)\left(y_{i}-\bar{y}\right)}{\sqrt{\sum_{i=1}^{n}{\left(x_{i}-\bar{x}\right)}^{2}}\sqrt{\sum_{i=1}^{n}{\left(y_{i}-\bar{y}\right)}^{2}}}$$

where n denotes the number of pairs of variables x and y , $x_{i}$ and $y_{i}$ denote the values of x and y , and $\bar{x}$ and $\bar{y}$ denote the means of x and y , respectively.

### 2.7 Proposed stacking ensemble learning framework

**FIGURE 2:**
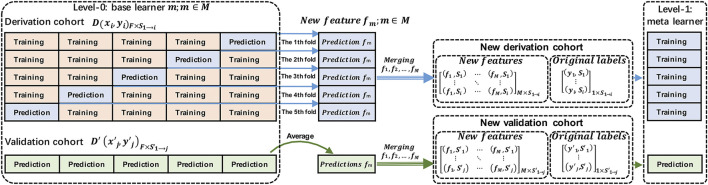
Proposed architecture of stacking with stratified five-fold cross-validation.

Figure 2 presents the architecture of stacking with stratified five-fold cross-validation. The derivation cohort ( D ) and the validation cohort ( $D^{\prime }$ ) were used for each base learner, and both consisted of feature vectors (i.e., $x_{i}$ and $x_{j}^{\prime }$ , respectively) and the corresponding original labels (i.e., $y_{i}$ and $y_{j}^{\prime }$ , respectively). F denotes the number of input features, and $S_{1\to i}$ and $S_{1\to j}^{\prime }$ denote the numbers of samples in the derivation cohort and the validation cohort, respectively.

The following steps were used to implement stacking (Kalagotla et al., 2021):

Step 1: Split the derivation cohort into five stratified folds. For each base learner m , use the first four folds for training and the rest for testing. Repeat this five times. Finally, take all out-of-fold predictions together as a new feature $f_{m}$ for fitting. Calculate the average value of predictions made by the base learner m in the validation cohort for each fold, and set this as a new feature $f_{m}$ for testing.

Step 2: Repeat step 1 for all base learners M ( M = 5 in this study). Merge all the new features (i.e., $f_{1},\;f_{2}$ , … , and $f_{M}$ ) created over the datasets D and $D^{\prime }$ . The new dataset then contains M attributes. Keep the original labels as the labels of this dataset.

Step 3: Select a model as a meta-learner (a linear regression model in this study). Generate this by combining the predictions made by the base learners to summarize the correct information. Train the meta-model and evaluate it in the final, new, derivation cohort and the new validation cohort.

### 2.8 Comparison of performance of models

#### 2.8.1 Evaluation of imputation and stacking

Given that multiple important features included in the stacking model had some missing data points—for example, the variable for ALT had 1,021 missing values—we performed imputation evaluation by reconstructing the prediction models such that the missing data points in the optimal feature subset were omitted, rather than imputed, to show whether imputation had generated a bias. First, an omitted dataset derived from the original dataset with the optimal feature subset, but with the missing data points omitted, was generated. The omitted dataset was split and data preprocessing was performed on it according to the aforementioned methods. Second, the performance of the generated base regression models was compared by using the two datasets, i.e., the transformed dataset and the omitted dataset, both of which had the same optimal feature subset. Third, the hyperparameters for each base model were adjusted to yield better predictive performance *via* the grid search method in scikit-learn. It evaluated the given hyperparameter combinations of the model by using “neg_mean_absolute_error” as the evaluation metric and 10-fold cross-validation (Radzi et al., 2021). This technique allows inexperienced data scientists to acquire recommendations for tuning the parameters, but may be time consuming and inefficient in case of a large number of parameters. Finally, the performance of the proposed stacked ensemble model after the optimization of the base models was compared with that of the base models before optimization.

#### 2.8.2 Evaluation metrics

Aside from the MAE, the following evaluation metrics were used to quantitatively evaluate the performance of the models: R-square ( $R^{2}$ ), mean squared error (MSE) (Cesar de Azevedo et al., 2021), mean relative error (MRE) (%) (Zhu et al., 2021a), and ideal rate (IR) (%) (Guo et al., 2021). These indices were calculated as follows:

$$R^{2}=1-\frac{\sum_{i=1}^{n}{\left({{\hat{y}}_{i}-y}_{i}\right)}^{2}}{\sum_{i=1}^{n}{\left({\hat{y}}_{i}-{\bar{y}}_{i}\right)}^{2}}$$

$$MSE=\frac{1}{n}\;\overset{n}{\underset{i=1}{\sum }}{\left(y_{i}-{\hat{y}}_{i}\right)}^{2}$$

$$MRE\;\left(\%\right)=\frac{1}{n}\;\overset{n}{\underset{i=1}{\sum }}\frac{\left(y_{i}-{\hat{y}}_{i}\right)}{{\hat{y}}_{i}}\times 100\%$$

$$IR\;\left(\%\right)=\frac{Number\;of\;predicted\;TDM\;within\pm 30\%\;of\;actual\;TDM\;}{Total\;number\;of\;actual\;TDM}\times 100\%$$

where $y_{i}$ , ${\hat{y}}_{i}$ , and ${\bar{y}}_{i}$ are the predicted, measured values, and the mean values, respectively.

### 2.9 Model interpretability

Ensemble learning models are in general criticized as “black-box” models (i.e., they are not interpretable) due to their complexity. Furthermore, the good performance of a model typically does not mean that its predictions are always correct. Therefore, in AI-assisted clinical decisions, medical workers usually ask such questions as “why should we trust the predictions of black-box models?” and “what are the mechanisms behind the model’s predictions?” There is thus a need to solve the problem of model interpretability, the concept of which involves the intuition behind the predictions of the model, that is, the relationships between the inputs and the outputs (Linardatos et al., 2020).

#### 2.9.1 Local interpretable model-agnostic explanations (LIME)

LIME is a perturbation-based method that was proposed by Ribeiro et al. (2016) for the concrete implementation of local surrogate models that are used to explain individual predictions of black-box models. This technique mixes perturbed inputs and the corresponding outputs of black-box models to generate a new dataset weighted around the instance being explained. In the perturbed dataset, an interpretable model is first trained, and the learned model then approximates the key features by generating their contributions to the outcomes of predictions in individual instances. Mathematically, the explanations produced by the LIME method can be expressed as follows:

$$\zeta \left(x\right)=arg\min_{g\in G}L\left(f,\;g,\pi_{x}\right)+\Omega \left(g\right)$$

where ζ(x) represents local surrogate models with the interpretability constraint, g denotes an explanation model (e.g., decision trees or linear regression models) for instance x that minimizes the loss function L (e.g., binary cross-entropy or mean squared error) that measures the discrepancy between the model g and the original model f (e.g., an XGBoost model) in the neighborhood $\pi_{x}$ (i.e., the proximity measure defining the extent of the locality around instance x ), G is the collection of all possible explanations, and Ω(g) is a penalty for the model complexity.

LIME supports explanations for structured datasets (e.g., tabular) and unstructured datasets (e.g., image and text), covering regression and classification models. For the regression and classification models that use tabular (i.e., matrix) data, the interpretations of LIME results are similar. LIME works internally as follows:

1) Given an observation, generate a fake dataset based on it with slight value modifications, and then permute the fake dataset.
2) Compute distance metrics (or similarity metrics) between original observations and permuted fake data. The similarity $\pi_{x}\left(z\right)$ is calculated as:

$$\pi_{x}\left(z\right)=\exp\left(-\frac{D{\left(x,z\right)}^{2}}{\sigma^{2}}\right)$$

3) Apply our original complex ML model to make a prediction on this new permuted fake data.
4) Select M number of features that best describe our complex ML model’s performance on permuted fake data.
5) Fit a simple model (e.g., linear or logistic regression) to the permuted fake data, explaining the complex model outcome with M features from the permuted fake data weighted by its similarity to the original observation.
6) Use the resulting feature weights derived from that simple model to explain local behavior.

We used the “LimeTabularExplainer” class and the “explain_instance ()” function available from the “lime_tabular” module in the LIME algorithm to observe two prediction-related behaviors of the stacking model by using representative drawings of two samples from the validation cohort. In this study, we specified the parameter attributes of “mode” and “feature_names” in the “LimeTabularExplainer” class to be regression and the list of feature names of the training data, respectively. Besides, we set the random_state parameter values to 0, 1, and 10, to observe the stability of model interpretation. Default settings were used for some other important parameters of this class, including “kernel_width (None),” “kernel (None),” and “feature_selection (auto).” Some important parameters of the “explain_instance ()” method also retained their default settings, including “model_regressor (None, i.e., defaults to ridge regression),” “distance_metric (euclidean),” “sampling_method (gaussian),” and “num_samples (5000).”

#### 2.9.2 Partial dependence plot (PDP)

Unlike LIME, the PDP is a global model-agnostic method. It shows the marginal effect of one or two features on the model (Friedman, 2001). A PDP tells us whether a linear, monotonic, or more complex relationship between the predicted response and a feature exists by considering all instances. This method of global interpretation can also explore how these features interact, whereby the degree of variation in the predicted outcome may be measured. The partial dependence function for regression can be described as follows:

$$\hat{f_{s}}\left(x_{s}\right)=E_{x_{c}}\left[\hat{f}\left(x_{s},x_{c}\right)\right]=\int \hat{f}\left(x_{s},x_{c}\right)dP\left(x_{c}\right)$$

where $x_{s}$ denotes the set of features of interest for which the function above should be plotted, $x_{c}$ denotes the complement set that contains all other features used in the ML model $\hat{f}$ , and dP denotes the marginal distribution. The union of two feature vectors $x_{s}\cup x_{c}$ is the total feature space x . The function $\hat{f_{s}}\left(x_{s}\right)$ is then estimated by calculating averaging predictions with actual feature values of $x_{c}$ in the training data at given values of $x_{s}$ . This is also known as the Monte Carlo method:

$$\hat{f_{s}}\left(x_{s}\right)\approx \frac{1}{n}\overset{n}{\underset{i=1}{\sum }}\hat{f}\left(x_{s},x_{c}^{i}\right)$$

where n denotes the number of instances in the dataset and $x_{c}^{i}$ denotes all observations from the dataset of features that are not of interest to us. Finally, the PDP visualizes the averaged relationship between the features and the predicted outcome, which is conventionally displayed by a trend line. In this study, we used the sklearn.inspection module to create one-way and two-way PDPs.

### 2.10 Software and implementation

All ML models were constructed by using the scikit-learn (version 0.23.2, https://scikit-learn.org/stable/index.html) and XGBoost (version 1.3.3, https://xgboost.readthedocs.io/en/latest/) packages in Python (version 3.8.5, https://www.python.org) (Pedregosa et al., 2011; Chen and Guestrin, 2016). All statistical analyses and visualizations were implemented by using packages of pandas (version 1.1.3, https://pandas.pydata.org), numpy (version 1.19.2, https://numpy.org), scipy (version 1.5.2, https://scipy.org), matplotlib (version 3.3.2, https://matplotlib.org), seaborn (version 0.11.0, https://seaborn.pydata.org), missingno (version 0.5.0, https://libraries.io/pypi/missingno), and lime (version 0.2.0.1, https://libraries.io/pypi/lime). All experiments were performed in the Jupyter notebook (version 6.1.4, https://jupyter.org) launched by Anaconda software (version 1.10.0, https://www.anaconda.com).

## 3 Results

### 3.1 Dataset overview

Table 2 summarizes the 472 features (303 categorical features and 169 continuous features) of the complete original dataset. They included general patient information, information relating to substance abuse, history of diagnosis and disorders, blood types, phenotypes, genotypes and gene polymorphisms, dosage regimens, combined drugs, and the results of TDM measurements of other medications and biochemical analyses when OLZ concentrations were simultaneously determined.

**TABLE 2:** A summary of features in the original dataset.

Items	Features
General patient information (four features)	Gender, age, body weight (abbreviation: BW), height
Substance abuse (three features)	Smoking history, drinking history, history of other substance abuse
Diagnosis and disorder history (three features)	Diagnosis of schizophrenia, diagnosis of bipolar affective disorder, allergic history
Blood types (two features)	ABO blood type, Rh blood type
Phenotypes, genotypes, and gene polymorphisms (13 features)	*MTHFR (C677T)* polymorphism, *MTHFR* phenotype, HLA-B*1502 genotype, *CYP2C19* genotype, *CYP2C19* phenotype, *CYP2D6* genotype, *CYP2D6* phenotype, *CYP2D6 (G4180C)* polymorphism, *CYP2D6 (G2988A)* polymorphism, *CYP2D6 (C2850T)* polymorphism, *CYP2D6 (G1846A)* polymorphism, *CYP2D6 (C100T)* polymorphism, *ApoE* genotype
Dosage regimens (three features)	Daily dose of OLZ [abbreviation: daily dose (OLZ)], dosage regimen of OLZ, daily dose frequency of OLZ
Combined drugs (280 features)	Valproic acid, risperidone, rifampicin, warfarin, clozapine, sertraline, fluvoxamine, perphenazine, carbamazepine, fluoxetine, etc.
Results of TDM measurements of other medications (24 features)	Concentrations of valproic acid [abbreviation: C (Valproic acid)], sertraline, fluoxetine, fluvoxamine, risperidone, oxcarbazepine, venlafaxine, clozapine, lamotrigine, perphenazine, aripiprazole, etc.
Information relating to biochemical analyses (140 features)	Time of TDM blood sampling after first administration of OLZ (abbreviation: time of blood sampling after first administration), alanine transaminase (abbreviation: ALT), serum potassium (abbreviation: K), serum sodium (abbreviation: Na), absolute monocyte count (abbreviation: MONO#), white blood cell count (abbreviation: WBC), red blood cell count (abbreviation: RBC), serum creatinine (abbreviation: Cr), uric acid (abbreviation: UA), creatine kinase (abbreviation: CK), C-reactive protein (abbreviation: CRP), total cholesterol (abbreviation: TC), etc.

**FIGURE 3:**
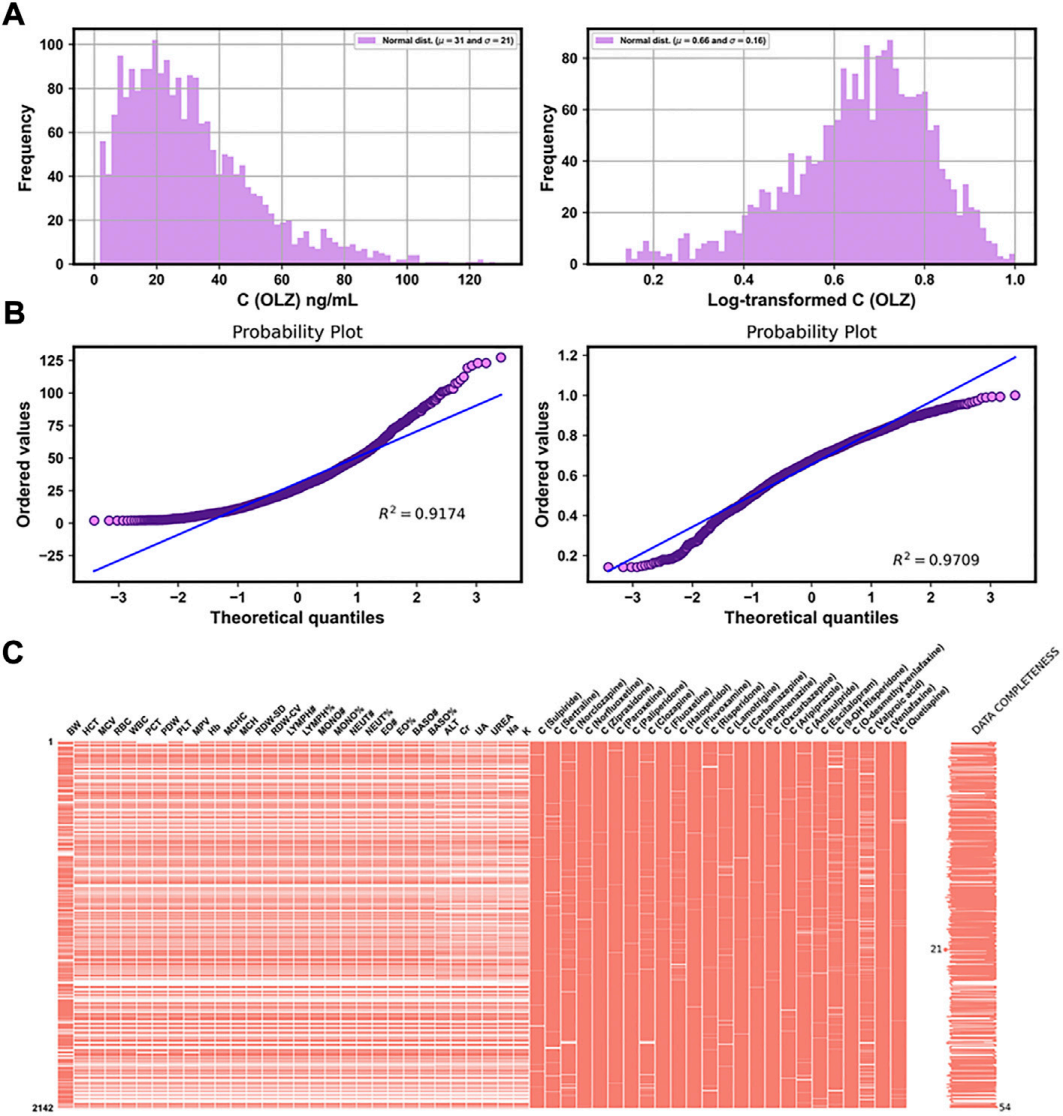
Frequency histograms **(A)** and Q–Q plots **(B)** of C (OLZ) and the log-transformed C (OLZ). **(C)** Chart of the matrix of missing data for 54 features, with fewer than 50% missing values in the original dataset.

Table 3 shows the distributions of the labels and the partial features in the entire original dataset, respectively. Figures 3A,B show comparisons of the frequency histograms and quantile–quantile (Q–Q) plots of the C (OLZ) and the log-transformed C (OLZ), indicating that log transformation tended to make the distributions more symmetric and normal. The data were rescaled to the range from 0.143 to 1.000.

**TABLE 3:** Distribution of partial continuous and categorical data in the original dataset (*n* = 2,142).

Continuous data	Values [median (min–max)]	Missing [n (%)]	Categorical data	Values	Distribution [n (%)]
C (OLZ) (ng/ml)	26.43 (2.00–127.31)	0 (0%)	Gender	Male	1235 (57.66%)
Age (years)	47 (12–94)	0 (0%)		Female	907 (42.34%)
BW (kg)	61 (26–121)	462 (21.57%)	Smoking history	Yes	308 (14.38%)
Daily dose of OLZ (mg)	15 (1.25–30)	0 (0%)		No	1347 (62.89%)
Time of TDM blood sampling after first administration of OLZ (days)	18 (0–483)	0 (0%)		Unknown	487 (22.73%)
ALT (U/L)	18 (3–632)	1021 (47.67%)	Diagnosis of schizophrenia	Yes	999 (46.64%)
Na (mmol/L)	140.3 (121.0–150.0)	1046 (48.83%)		No	1143 (53.36%)
K (mmol/L)	3.99 (2.20–5.21)	1046 (48.83%)	Co-administration of valproic acid	Yes	778 (36.32%)
Cr (μmol/L)	70 (28–203)	1067 (49.81%)		No	1364 (63.68%)
MONO# (×10^9^/L)	0.49 (0.07–2.07)	985 (45.99%)			
C (Valproic acid) (mg/L)	0.00 (0.00–150.00)	231 (10.78%)			

Of the 169 continuous features, 54 with fewer than 50% missing values were reserved, and are illustrated in Figure 3C. This was in addition to four features that did not contain any missing values (see Table 3). The missing values of features are shown in Figure 3C by white lines in each column of the matrix chart with sparklines to the right, indicating the completeness of the data. Missing value imputation and min–max normalization were then applied to these continuous features.

By dropping the first column after one-hot encoding on the 303 categorical features of the original dataset, the number of categorical variables increased to 389. A new, transformed, dataset containing 58 continuous features and 389 categorical features was created.

### 3.2 Base regression models

Table 4 shows the MAE of the predictions by the candidate regression models of the log-transformed C (OLZ) on the derivation cohort of the entire transformed dataset. An overall comparison of these models in the test set indicated that most ensemble learning models were superior to single ML models. The top five regression models: the ETR, XGBR, RFR, BR, and GBR, were chosen as base models for stacking.

**TABLE 4:** An overall comparison of the candidate models in terms of the MAE at a 95% confidence interval (CI) in the derivation cohort of the transformed dataset.

Candidate models	MAE in the training set	95% CI of MAE in the training set (+/-)	MAE in the test set	95% CI of MAE in the test set (+/-)
ETR	2.005×e^−7^	1.773×e^−7^	0.060	0.011
XGBR	0.008	0.001	0.066	0.011
RFR	0.025	0.001	0.066	0.008
BR	0.028	0.001	0.071	0.011
GBR	0.059	0.001	0.071	0.009
SVR	0.063	0.001	0.076	0.009
KNR	0.067	0.001	0.086	0.016
DTR	2.005×e^−7^	1.773×e^−7^	0.086	0.013
ABR	0.080	0.003	0.086	0.010
MLR	0.055	0.001	2.767×e^+8^	5.208×e^+8^

### 3.3 Best feature combination for prediction

**FIGURE 4:**
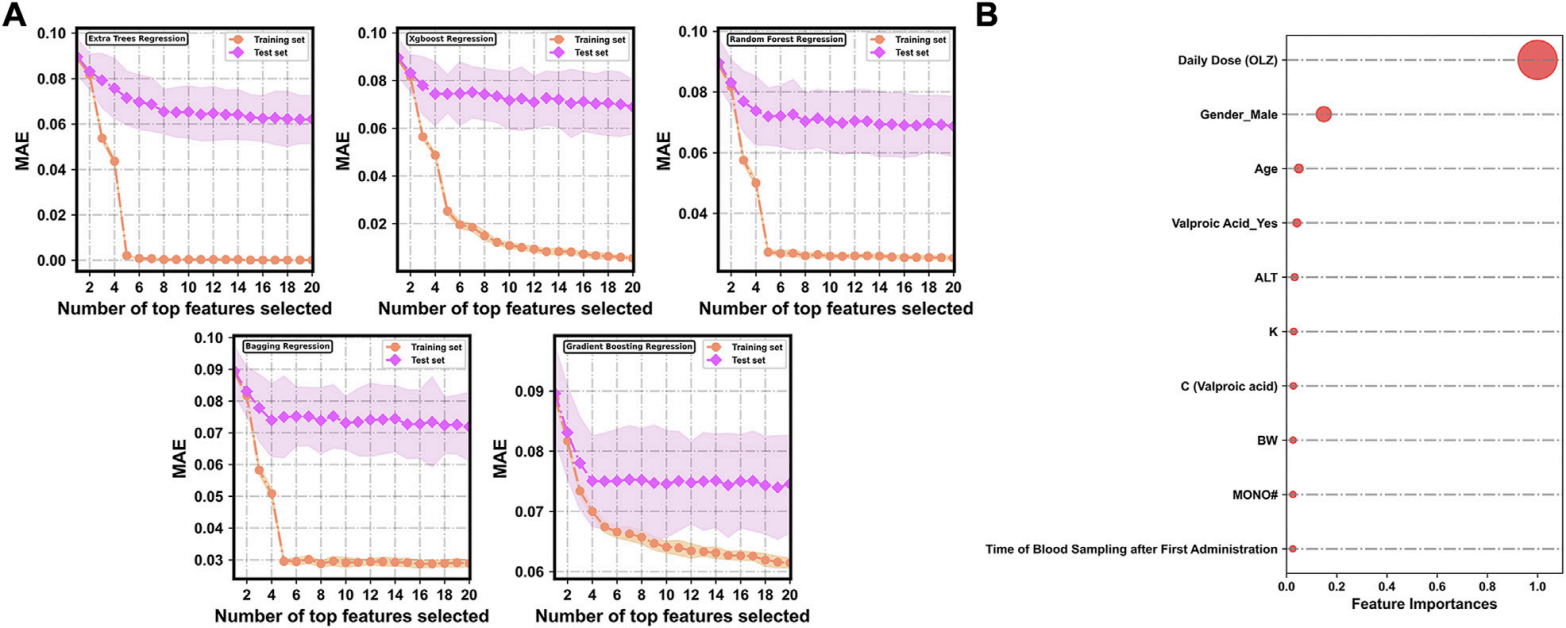
**(A)** Evolution of prediction errors for various compositions of feature subsets selected by the random forest-based sequential forward feature selection strategy. The corresponding 95% CIs of the MAE obtained by 10-fold cross-validation are represented by the colored areas. **(B)** Relative feature importance of the top 10 features.

Figure 4A shows changes in the predictive performances of the base regression models with various compositions of feature subsets selected by the random forest-based sequential forward feature selection strategy. A flexible and simple model generally has as few features as possible but delivers the best possible predictive performance. To discard irrelevant features, we identified the point (corresponding to the top 10 features selected) at which no considerable reduction occurred in the MAE in the test sets of all the base models when extra features were added to them. The top 10 features were ranked in terms of relative importance as follows (Figure 4B): daily dose (OLZ) (1.0000), gender_male (0.1484), age (0.0491), valproic acid_yes (0.0414), ALT (0.0323), K (0.0288), C (valproic acid) (0.0273), BW (0.0262), MONO# (0.0255), and time of blood sampling after first administration (0.0251).

**FIGURE 5:**
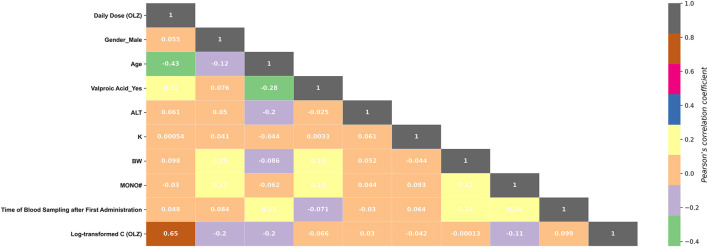
Heatmap of the Pearson correlations between the log-transformed C (OLZ) and the finally selected features.

Among these 10 features, valproic acid_yes and C (valproic acid) were found to be multi-collinear (Pearson *r* = 0.801, *p* < 0.001). As such, only the more important feature, “valproic acid_yes,” was retained in the optimal feature subset. Figure 5 shows the correlations between the log-transformed C (OLZ) and the finally selected features. The daily dose (OLZ) was identified as a key positive factor associated with the log-transformed C (OLZ), showing a moderate correlation coefficient of 0.65. The other features showed either weak positive correlations (e.g., ALT and time of blood sampling after first administration) or weak negative correlations (e.g., gender_male, age, valproic acid_yes, K, BW, and MONO#) with the log-transformed C (OLZ).

### 3.4 Performance analysis of models

**FIGURE 6:**
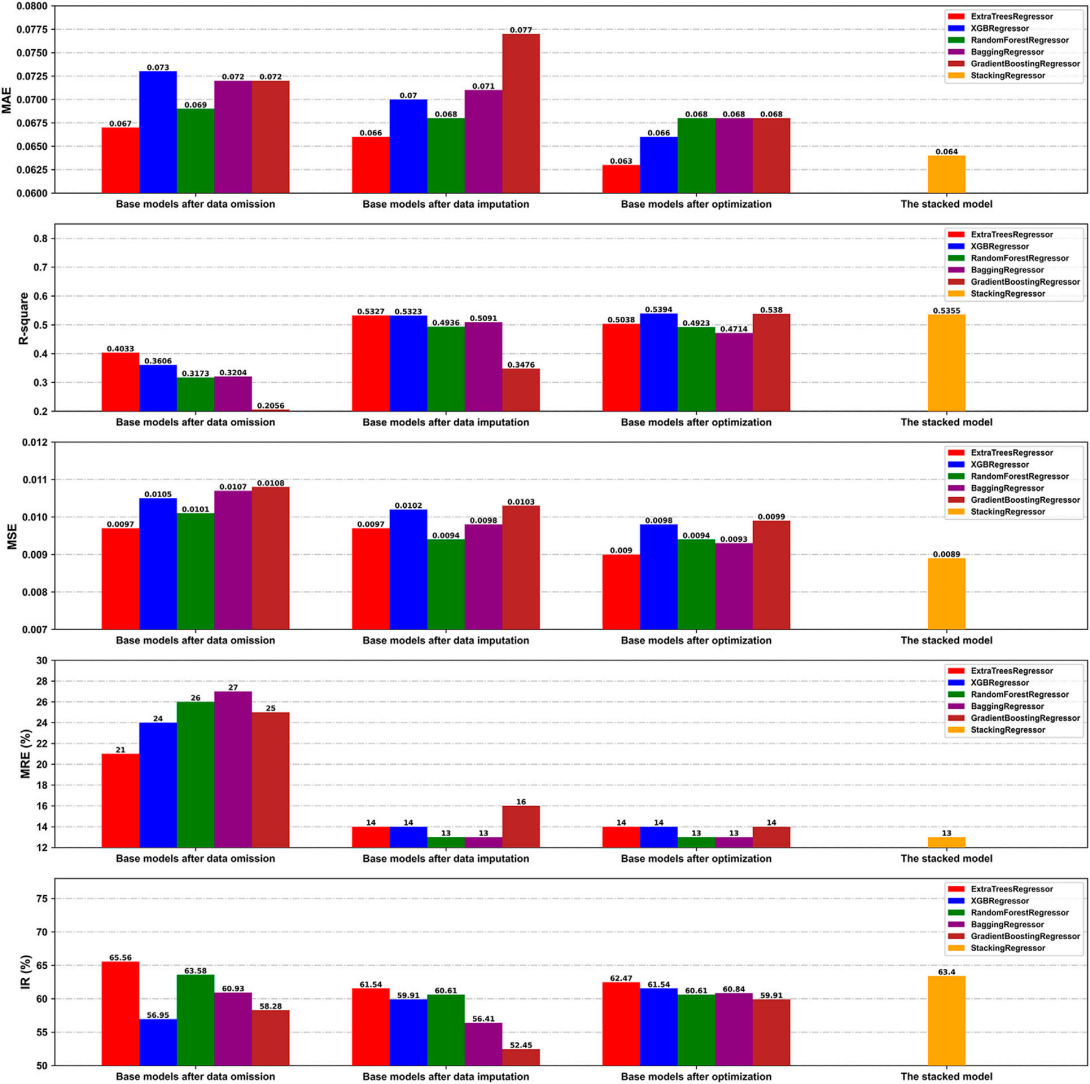
Comparison of the prediction performance of our models on the validation cohorts under different conditions in terms of the MAE, *R* ^2^, MSE, MRE, and IR.

The omitted dataset was generated in a 752 × 10 matrix format to evaluate the influence of kNN imputation of the missing values of the optimal feature subset on the performance of the model. Based on a ratio of division of 8:2, the omitted dataset was divided into the derivation and the validation cohorts, containing 601 and 151 samples, respectively. Furthermore, the predictive performances of the base models after data omission and imputation were compared on the respective validation cohorts (*n* = 151 and *n* = 429, respectively). As is shown in Figure 6, the base models after data imputation on the whole generated values of the MAE and MSE similar to, but those of $R^{2}$ and MRE better than, those of the models after data omission. Thus, the imputed dataset was considered during modeling owing to its larger number of samples.

We also compared the performance of the base models before and after hyperparameter optimization on the validation cohort of the transformed dataset. Table 5 presents the optimized hyperparameters of each base model. Overall, the optimal base models exhibited enhanced generalization performance by reducing overfitting, as illustrated by the improvements in several indices such as the MAE and IR (Figure 6).

**TABLE 5:** Optimized hyperparameters of each base model.

Base model	Hyperparameters
ETR	‘n_estimators’: 251, ‘max_depth’: 30, ‘min_samples_leaf’: 1, ‘min_samples_split’: 2, ‘max_features’: sqrt
XGBR	‘n_estimators’: 271, ‘max_depth’: 8, ‘min_child_weight’: 4, ‘gamma’: 0, ‘colsample_bytree’: 1.0, ‘subsample’: 1.0, ‘learning_rate’: 0.19
RFR	‘n_estimators’: 102, ‘max_depth’: 23, ‘min_samples_leaf’: 1, ‘min_samples_split’: 2
BR	‘n_estimators’: 106, ‘max_features’: 0.9, ‘max_samples’: 1.0
GBR	‘n_estimators’: 178, ‘max_depth’: 8, ‘min_samples_leaf’: 1, ‘min_samples_split’: 3

**FIGURE 7:**
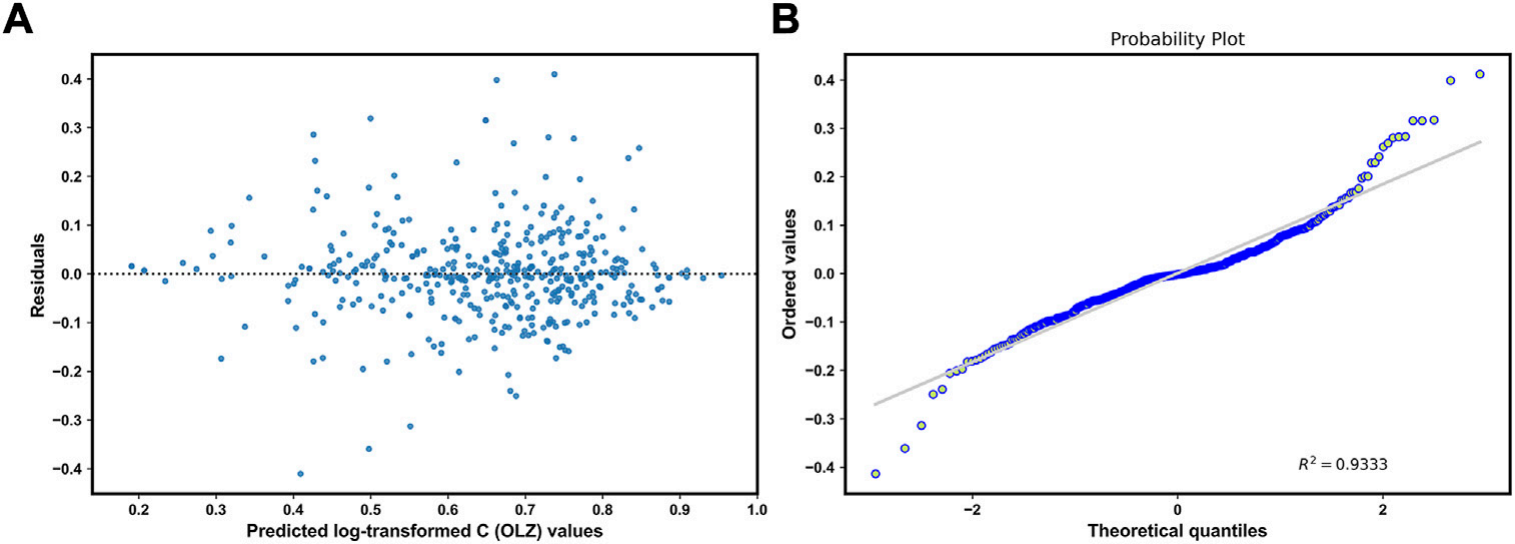
Residuals plots: Plot of residuals versus the predicted values **(A)**, and normal plot of the residuals **(B)**.

Finally, the state-of-the-art proposed stacking model provided an overall better performance than the other optimal base models, with MAE, $R^{2}$ , MSE, MRE, and IR values of 0.064, 0.5355, 0.0089, 13%, and 63.4%, respectively (Figure 6). Moreover, Figure 7 illustrates no clear patterns, and shows that the residuals were symmetrically distributed, which satisfies the assumption of a normal distribution. This shows that the proposed stacking model is appropriate and a good fit for the data.

**FIGURE 8:**
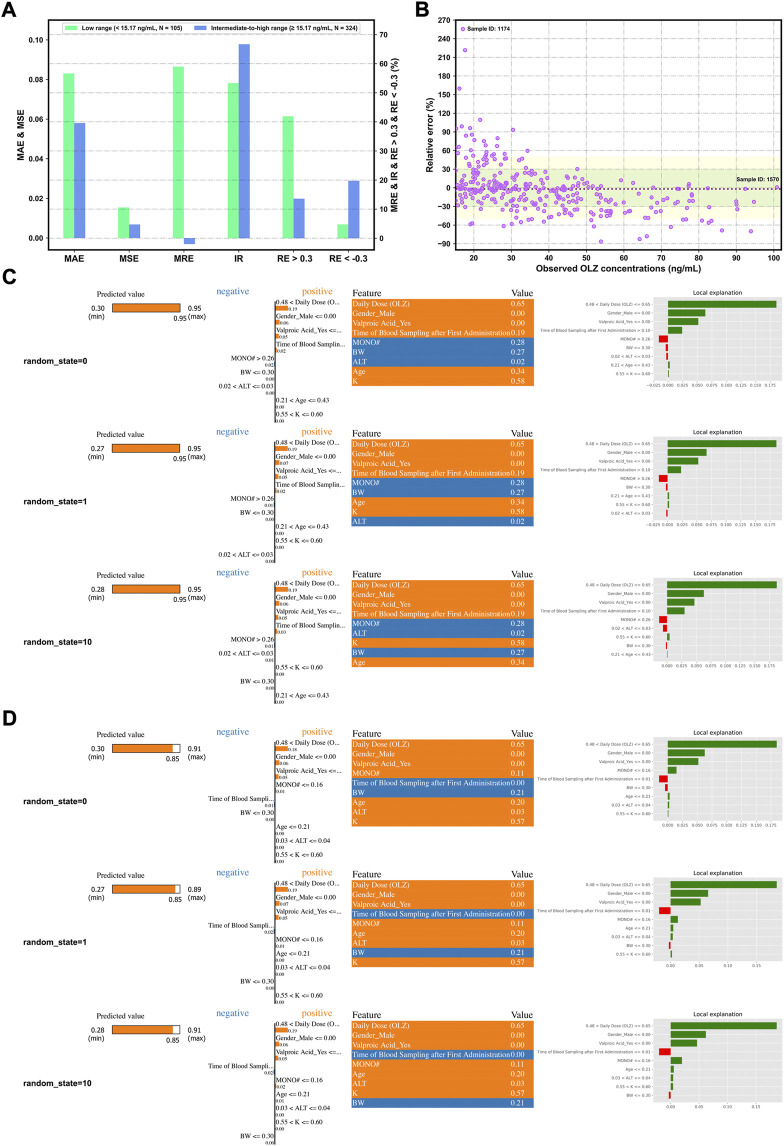
Assessing the forecasting performance of the proposed stacking model in terms of different ranges of C (OLZ) on the validation cohort: Histograms of various metrics in the context of the low and intermediate-to-high ranges **(A)**, and a scatterplot of the relative error (RE)% versus the observed C (OLZ) in the intermediate-to-high range **(B)**, where the red dotted lines denote the MRE, the colored areas denote the ±30% (green color) and ± 50% (yellow color) ranges of the RE, and the dotes labeled by sample ID 1174 and ID 1570 represent the maximum RE of prediction and the maximum observed C (OLZ), respectively. Interpretation of the results of prediction of samples ID 1174 **(C)** and ID 1570 **(D)** by the LIME algorithm using different random_state values. The four views for each sample, from left to right, show the predicted values of the explanation and the stacking models, the feature coefficients (the orange and blue colors depict positive and negative relationships, respectively), the feature values in this sample, and the local explanation plot of these features.

Notably, Figures 8A,B demonstrate more accurate predictions in the intermediate-to-high range of C (OLZ) (≥15.17 ng/ml; i.e., the 25% quartile of OLZ concentrations in the validation cohort) than in its low range (<15.17 ng/ml).

### 3.5 Interpretation of the proposed stacking model

Figures 8C,D show the explanation of decisions behind the predictions for the selected samples (ID 1174 and ID 1570) in terms of different random_state values. It shows significant differences in predictive performance in these two samples. To interpret these results, we conclude that relatively higher values of C (OLZ) can be attributed to the following reasons: 1) The high values of daily dose (OLZ) and time of blood sampling after first administration, 2) the lower value of MONO#, and 3) female patients (gender) or without the co-administration of valproic acid. The other factors, such as age, BW, and ALT, also affected the predictive behaviors of the model, whereby a large difference in performance on the above samples was noted. We reproduced similar results each time we reran the code with different random_state values for the same data sample, indicating that the explanations of our model were trustworthy.

**FIGURE 9:**
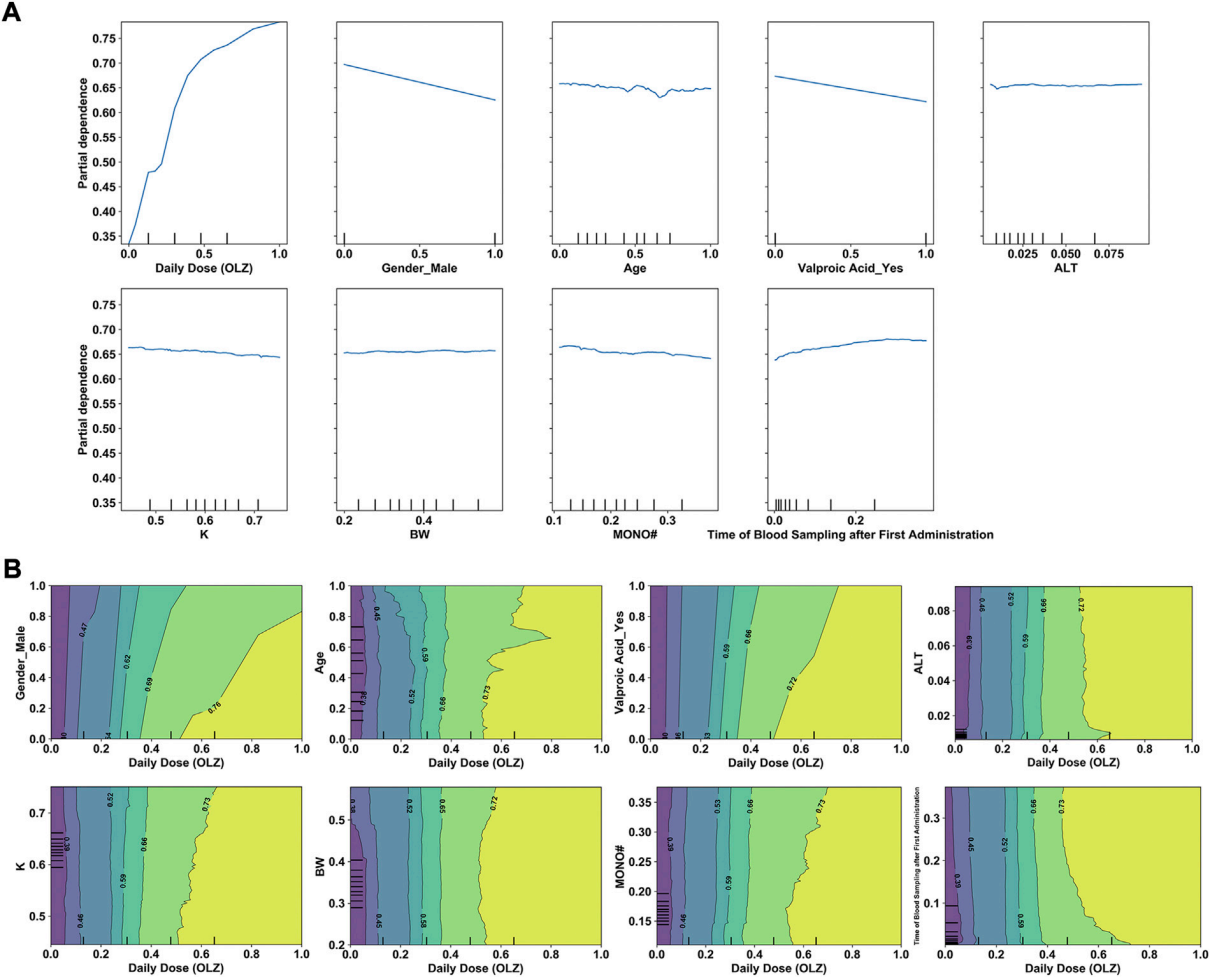
**(A)** One-way PDPs for features included in the stacking model. **(B)** Two-way PDPs of the interactions between the daily dose (OLZ) and other features.

Having determined the key features, we examined their effects on the predicted outcomes to improve our understanding of the use of ML-derived algorithms for the precision dosing process. Figure 9A shows the relationships between the selected features (*x*-axis) and log-transformed C (OLZ) (*y*-axis), revealing that changes in the normalized values of several continuous feature variables [e.g., the daily dose (OLZ) and age] exhibited non-linear relationships with changes in the log-transformed C (OLZ). Figure 9B shows the interactions between the daily dose (OLZ), as the most important feature, and the other features as well as their influence on our predictions. For example, for a normalized daily dose (OLZ) greater than 0.60, the log-transformed C (OLZ) in the elderly population was higher than that in child and adolescent populations. Within this range, it was nearly independent of the BW.

## 4 Discussion

Unlike previous studies that used only homogeneous ensembles (e.g., XGBoost) or simple weighted average ensembles for ML-assisted TDM (Zhu et al., 2021a; Guo et al., 2021; Hsu et al., 2021; Huang et al., 2021; Zheng et al., 2021; Lee et al., 2022), our is the first study, to the best of our knowledge, to explore the real-time estimation of drug concentrations by using a stacking ensemble framework as an MIPD tool. Our work here shows that stacking a heterogeneous ensemble, overall, is superior to homogeneous ensemble-based methods (e.g., bagging and XGBoost models) on several comparisons of model performance on the TDM-OLZ dataset. For example, the proposed stacking model yielded MSE and IR values of 0.0089 and 63.4%, respectively, which were comparatively better than that of any single base models. Furthermore, the model generated MAE, $R^{2}$ , and MRE values of 0.064, 0.5355, and 13%, respectively, which were close to those of the best-performing base models as filtered through the corresponding metrics, i.e., the ETR with an MAE of 0.063, the XGBR with a value of $R^{2}$ of 0.5394, and the RFR and the BR with an MRE value of 13%. Hence, the state-of-art stacking model outperformed the individual regressors, and can be considered to be the optimal model for predicting OLZ concentration. In this stacking model, the five top-ranking homogeneous ensemble models (i.e., ETR, XGBR, RFR, BR, and GBR) were used to construct the complex but diverse base models, also called strong learners. They were trained by using stratified five-fold cross-validation to ensure that all data in the derivation cohort participated in training to avoid overfitting (Wu et al., 2021; Sayari et al., 2022). A simple linear regression model was used as the meta-model in model stacking, and provided an intuitive and smooth interpretation of predictions made by the base regressors, thus further reducing the probability of overfitting (Zhang et al., 2021). Our study also demonstrated that the stacking model could integrate the complementary merits of the bagging and the boosting models, thus achieving stable and significantly enhanced predictions (Sesmero et al., 2015).

The interpretability of a model is vital for its clinical use. We have mentioned that the proposed stacking model obtained more “trustworthy” predictions in the intermediate-to-high range of the C (OLZ) (≥15.17 ng/ml). A much higher variance at a lower C (OLZ) was also noted, possibly due to the problem of overfitting in the training process and a greater influence of uncontrolled confounders, including errors in TDM measurement and the genetic effects of gene polymorphisms (e.g., polymorphisms in *CYP1A2*) associated with OLZ metabolism (Zhu et al., 2021a). Moreover, the analysis of relative feature importance revealed that daily dose (OLZ) was the most important feature, which was consistent with the findings of the PDPs, where the log-transformed C (OLZ) sharply increased with normalized daily dose (OLZ). The PDPs also revealed that a decreasing trend in the log-transformed C (OLZ) was associated with normalized age values ranging from zero to approximately 0.65, and a small increase was subsequently observed with an increase in the normalized age. Deng et al. (2020) have reported that patients younger than 60 years had higher C (OLZ) than patients older than 60 years. A previous study by An et al. (2021) also revealed that advanced age is related to a lower C (OLZ). However, another prior study arrived at the opposite conclusions. The authors there found that the effects of age on C (OLZ) became pronounced with advanced age. They noted that patients aged 70–79 years had the highest median measured C (OLZ), and concluded that patients older than 70 years should be subdivided and considered for dose reduction (Castberg et al., 2017). Our work, therefore, offers a new perspective on the explanation of these conflicting findings. Other features, such as male (sex) or co-administered valproic acid, were identified as having had a negative influence on the predicted values of the log-transformed C (OLZ), which aligns with previous reports (Bigos et al., 2008; Zang et al., 2021). The two-way PDPs showed that the interactions between the daily dose (OLZ) and most features were pronounced in case of a high normalized daily dose (OLZ); however, the interactional dependencies among them tended to not be prominent as the normalized daily dose (OLZ) was increased to a certain extent. To further explore the comprehensive effects of these features on predictions of the model, we used the LIME methodology to compare the predictive behaviors of the stacking model on two discrepant samples having the same feature values of the daily dose (OLZ), gender_male, and valproic acid_yes. The other indicators (e.g., time of blood sampling after first administration, age, and MONO#) profoundly affected the predictive accuracy of our model in this case. One possible explanation for this is that inadequately similar samples were learned by the model.

**FIGURE 10:**
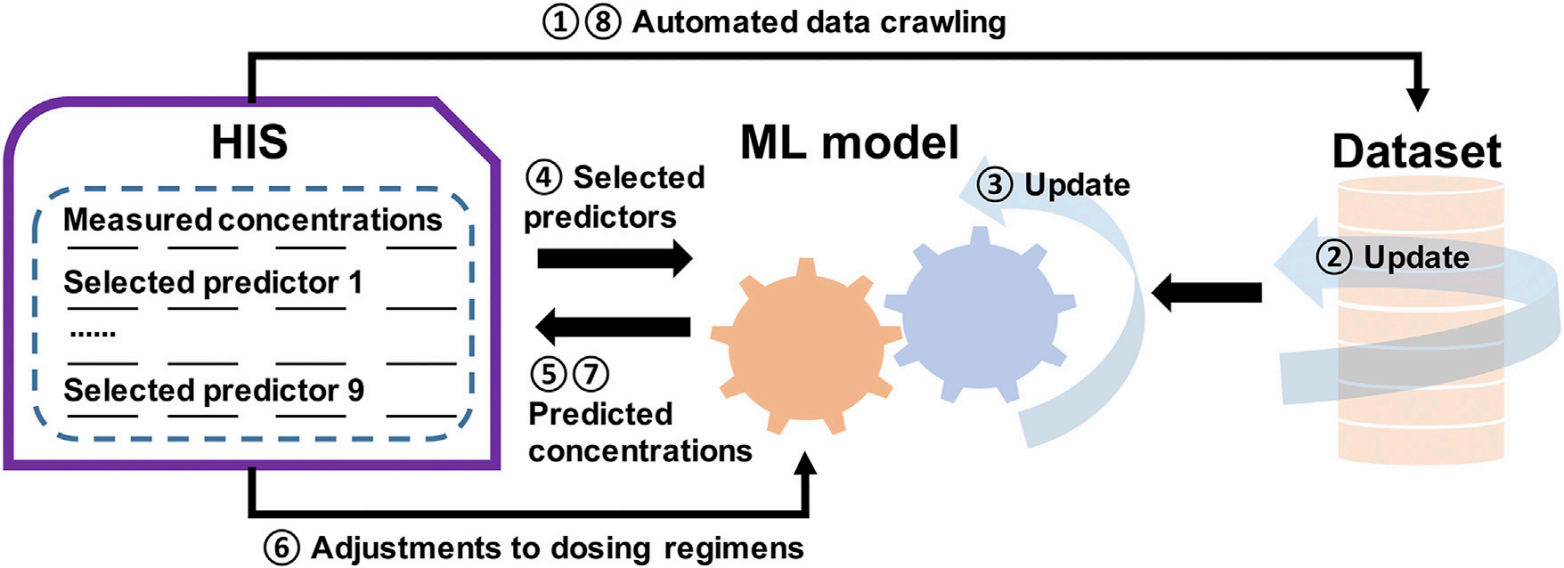
Illustration of a general framework of the self-learning and optimization processes of the ML model for a more precise, individualized dose of OLZ.

Our work also shows that the intersection between ML and pharmacometrics might have the potential to advance data sciences, as mentioned by Koch et al. (2020). For instance, our model discovered some confounding factors (e.g., MONO#, K, and ALT) that influenced the pharmacokinetics of OLZ, and this has not been reported in previous pharmacometric studies. This indicated that these factors could be used as potential covariates in future popPK studies to explore their impact. On the other hand, the updating in pharmacometrics is static which requires new models (McComb et al., 2022). However, as an extension of this work, we have designed an AI-assisted system to predict drug concentration, where the dataset is updated according to automated data crawling from the Hospital Information System (HIS), followed by a dynamically updated ML model to predict concentrations based on the selected predictors. The dosing regimens are then adjusted, and new measurements and predictions are performed. This process is recurrent, and improves the self-learning of the ML model (Figure 10).

This work also has certain limitations. First, some relevant characteristics of patients (e.g., polymorphisms in *CYP1A2*) were not included due to limitations of the test items. Previous studies have revealed a significant influence of polymorphisms in the *CYP1A2* gene on C (OLZ) and clinical outcomes (Czerwensky et al., 2015). However, a recent meta-analysis of the impact of *CYP1A2* genetic polymorphisms on the pharmacokinetics of CYP1A2-metabolized antipsychotic drugs did not confirm this association (Na Takuathung et al., 2019). More feature variables of patients, especially these controversial factors, should be considered in future ML modeling. Second, despite a lack of consensus on model validation methods, the comparison of the predictive performance of the proposed stacking model and the pharmacometrics-based models on the external validation dataset may be worthy of further study. Third, some features are known to have effects on the C (OLZ). For example, smokers have been reported to exhibit significantly lower steady-state plasma concentrations of OLZ and N-desmethyl-olanzapine, and clear OLZ 55% faster than non-smokers/past smokers (Bigos et al., 2008; An et al., 2021). However, smoking history was excluded in the optimal feature subset of our model, owing to the small size of samples. This led to a slight imbalance in the feature categories (e.g., the proportion of smoking history was only 14.38%), and they were trained using few iterations. Besides, the flexibility of the implementation of our ML may be hindered due to the single-center study. Therefore, more data collected from routine TDM should be considered in future work to construct a retrospective, multicenter, large-scale, high-quality TDM database to optimize the model. Finally, each method of interpreting ML has inherent limitations. For example, the LIME approach might result in unstable interpretations, and PDPs might not work well if features in the given subsets are strongly correlated with one another. Thus, there is the room for improvement in techniques to interpret the results of ML methods (Linardatos et al., 2020).

## 5 Conclusion

In this study, we proposed a stacking ensemble learning framework to improve predictions of drug concentrations. The novel two-layer stacking ensemble, consisting of the ETR, XGBR, RFR, BR, and GBR models as the first layer and a linear regression model as the second layer, was generated based on a TDM-OLZ dataset that we derived from real-world multi-dimensional EHR data. Overall, the state-of-art stacking ensemble learning model outperformed the base models in terms of the MAE, $R^{2}$ , MSE, MRE, and IR on the validation cohort. Moreover, specific and practical interpretations of the results of the proposed model were obtained by using the interpretable learning methods LIME and PDP. This can help advance clinical data analysis by partnering with pharmacometrics. In conclusion, our study demonstrated the promise of using a stacking ensemble learning framework to advance MIPD. However, the proposed methodology needs to be further developed.

## Data Availability

The original contributions presented in the study are included in the article/supplementary material, further inquiries can be directed to the corresponding authors.
